# MyD88 orchestrates fatty acid metabolism in tumor-associated macrophages and non-alcoholic fatty liver disease-related hepatocarcinogenesis

**DOI:** 10.3389/fimmu.2025.1589255

**Published:** 2025-09-23

**Authors:** Haiqiang Chen, Xuanxuan Yan, Zuyin Li, Zhenzhong Deng, Jianchun Gu, Fanxin Zeng, Zhao Li, Jinhua Zhang

**Affiliations:** ^1^ The College of Life Science and Bioengineering, Beijing Jiaotong University, Beijing, China; ^2^ Department of Hepatobiliary Surgery, Peking University People’s Hospital, Beijing, China; ^3^ Department of Oncology, Xinhua Hospital Affiliated to Shanghai Jiaotong University School of Medicine, Shanghai, China; ^4^ Department of Clinical Research Center, Dazhou Central Hospital, Dazhou, Sichuan, China

**Keywords:** macrophage, MyD88, NAFLD, HCC, SREBP1

## Abstract

**Introduction:**

Non-alcoholic fatty liver disease (NAFLD) is a growing cause of cirrhosis and hepatocellular carcinoma (HCC). Toll-like receptors (TLRs) and their adapter protein, myeloid differentiation factor 88 (MyD88), are activated in NAFLD and contribute to its development. However, the specific role of MyD88 in myeloid cells that regulate NAFLD-associated hepatocarcinogenesis remains unknown.

**Methods:**

We used a diethylnitrosamine/high-fat diet-induced HCC mouse model with selective deletion of MyD88 from myeloid cells to investigate NAFLD-HCC, and we evaluated the development of NAFLD-HCC histologically and genetically.

**Results:**

Myeloid cell MyD88-deficient (Lyz^MyD88−/−^) mice were protected from diet-induced obesity and developed fewer and smaller liver tumors. MyD88 deficiency in myeloid cells also attenuated macrophage M2 polarization and fat accumulation in HCC tissues. Mechanistically, the loss of MyD88 signaling specifically inhibited macrophage M2 polarization, with decreased metabolism in a SREBP1/STAT6 pathway-dependent manner. Furthermore, liver tumor growth was attenuated in mice treated with a SREBP1 inhibitor. High SREBP1 and CD163 expression in HCC was associated with shorter survival of patients with HCC. Thus, our results indicate that MyD88 in myeloid cells promotes NAFLD-related HCC progression and may be a promising therapeutic target for HCC treatment.

**Discussion:**

MyD88 in macrophages has a promotional role in NAFLD-associated HCC. MyD88 promotes macrophage M2 polarization, which enhances the progression of NAFLD to HCC by activating the SREBP1/STAT6 pathway. MyD88 in macrophages may be a potential therapeutic and/or preventive target for NAFLD-associated HCC.

## Introduction

Liver cancer is a leading cause of cancer deaths worldwide, ranking as the third most frequent cause of cancer-related deaths in 2022 (1). Hepatocellular carcinoma (HCC) is prevalent form of primary liver cancer. It is a complicated disease with multiple pathogenic mechanisms caused by a variety of risk factors, including hepatitis virus infection, chronic alcohol consumption, hypertension, nonalcoholic fatty liver disease (NAFLD), etc. Recent epidemiology studies have shown that the incidence of NAFLD-related liver cancer is increasing globally (2, 3).

NAFLD is a dysfunctional stress response caused by excessive supply of nutrients in the liver, which includes a series of pathological changes in the liver, ranging from a simple intrahepatic accumulation of fat (steatosis or nonalcoholic fatty liver, NAFL) to various degrees of necrotic inflammation (nonalcoholic steatohepatitis, NASH) (2).

The association between NAFLD and various metabolic syndrome diseases such as diabetes and systemic hypertension is bidirectional (4). Insulin resistance is considered an essential component of the pathogenesis of NAFLD (5). In approximately 20% of affected patients, NASH will evolve into fibrosis and cirrhosis and potentially into HCC over approximately 15 years (6). The NAFLD development process involves oxidative stress, inflammatory response, DNA damage and autophagy. Fat accumulation in the liver leads to cellular metabolic changes, causing potential harm and accelerating tumor progression (7, 8). Understanding the molecular mechanisms relevant to developing HCC related to NAFLD is crucial to developing more efficient targeted therapies for HCC.

The tumor microenvironment (TME) comprises the matrix in which tumor cells reside and plays significant roles in tumor occurrence, progression and drug resistance (9). One of its main features is the metabolic reprogramming of the TME (10). Tumor-associated macrophages (TAMs) are a major component of the TME and play a crucial role in tumor initiation, growth, invasion and metastasis. They exhibit different cellular phenotypes due to various micro-environmental stimuli, making them highly plastic (11, 12).

Recently, metabolic studies have revealed that specific metabolic pathways in macrophages are closely linked to their phenotype and function. Generally, pro-inflammatory macrophages (M1) primarily rely mainly on glycolysis and exhibit impairment of the tricarboxylic acid (TCA) cycle and mitochondrial oxidative phosphorylation (OXPHOS), whereas anti-inflammatory macrophages (M2) depend more on mitochondrial OXPHOS (13, 14). Studies have shown that macrophages are the most metabolically active cells consuming the most glucose in TME (15). Macrophage metabolism is regulated by a variety of signaling pathways, including HIF, PI3K/AKT, PPAR and AMPK pathways, which play key roles in macrophage polarization (16). Fatty acid oxidation (FAO) is critical for M2 macrophages polarization, and inhibiting FAO can promote for M2-to-M1 macrophage conversion (17–19). A better understanding of the mechanisms underlying the regulation of TAM metabolism and function may facilitate the development of more targeted and effective immunotherapies.

Toll-like receptors (TLRs) are innate immune receptors that detect pathogen-associated molecular patterns (PAMPs) and activate innate and adaptive immune pathways directly or indirectly. Myeloid differentiation factor 88 (MyD88) is a widely expressed cytoplasmic adapter protein, that transmits signals from members of both the TLR (excluding TLR3) and IL-1 receptor families (20). MyD88 is associated with hepatic metabolism. Hepatocyte-specific knockout of MyD88 promotes lipid accumulation and immune cell infiltration. In HFD-induced NASH models, deficiency of MyD88 in myeloid cells attenuates immune cell infiltration and lipid accumulation (21). MyD88 expression is significantly upregulated in liver tumor tissues compared to adjacent non-tumor tissues, and its elevated expression is associated with poor patient prognosis. In a DEN-induced murine hepatocellular carcinoma model, Kupffer cells were shown to promote IL-6 production in a MyD88-dependent manner, thereby driving hepatocarcinogenesis (22). Previous studies have found that MyD88 plays important roles in a variety of cancers. Mice lacking MyD88 developed fewer tumors than wild-type (WT) mice in azoxymethane (AOM)-induced intestinal tumorigenesis or diethylnitrosamine (DEN) - high fat diet-induced HCC (22–24).

MyD88 is expressed by various immune and non-immune components with varied roles depending on the cell type and circumstances. Recently, we have shown that MyD88 facilitates HCC development in myofibroblasts by boosting the production of CCL20 and promoting aerobic glycolysis (25). Hepatocyte-specific knockdown of MyD88 promotes fat accumulation, liver inflammation, and poor glucose tolerance (26). Macrophage metabolism is reprogrammed by TLR-dependent promotion of fatty acid synthesis via MyD88 (27). It is unknown whether and how MyD88 in macrophages regulates HCC.

This study investigated the role of TLR signaling in macrophages during NAFLD-related HCC using mice bred to demonstrate myeloid cell MyD88 deletion. Our findings showed that MyD88 deletion in myeloid cell reduced fat accumulation and tumor incidence in HFD-induced NAFLD and DEN/HFD-induced liver cancers. Moreover, we found that MyD88 in macrophages promoted macrophage M2 polarization through SREBP1/STAT6 pathway, enhancing NAFLD-related HCC development.

## Materials and methods

### Mice

MyD88^flox/flox^ (MyD88^fl/fl^) and Lysm-cre mice on a C57BL/6 background have been previously described (28, 29). Mice with a conditional knockout of MyD88 in myeloid-cell (Lyz^MyD88−/−^) were generated by crossing MyD88^fl/fl^ and Lysm-cre mice. Genotyping was performed as described previously (29). Control mice were cre-negative littermates. All mice were maintained in specific pathogen-free and humidity and temperature-controlled microisolator cages with a 12 h light/dark cycle in the Institute of Biophysics, Chinese Academy of Sciences. Lyz^MyD88−/−^ mice and their littermates used for the experiments were 6 to 8 weeks old. All animal studies were performed after being approved by the Institutional Laboratory Animal Care and Use Committee of Beijing Jiaotong University.

### DEN/HFD-induced hepatocellular carcinoma

The male mice were i.p. injected with DEN (50 μg/g body weight) (Sigma-Aldrich, St. Louis, MO, USA) at the age of 15 days and were fed HFD (60% of total energy from fat, 20% protein, 20% carbohydrate, Huafukang, Beijing, CN) beginning at 6 weeks of age. Liver tumorigenesis was monitored for 10 months.

### Blood biochemical assays

Blood samples of mice were centrifuged at 3000 rpm for 8 min to obtain serum. The levels of serum alanine aminotransferase (ALT), triglyceride (TG) and total cholesterol (TC) were detected by Beijing Vital River Laboratory Animal Technology (Beijing, China).

For the glucose tolerance test (GTT), blood samples were obtained at 0, 15, 30, 60 and 120 min after intraperitoneal injection of 2 g/kg dextrose. Blood glucose values were determined using Accu-Chek Performa glucometer (Roche, Basel, Switzerland).

### Histology and immunostaining

The sliced liver paraffin sections were stained with hematoxylin and eosin (H&E) (Zhongshanjinqiao, Beijing, China). To detect hepatic fat accumulation, cryostat liver sections were stained with Oil Red O (Baso, Zhuhai, Guangzhou, CN). For fluorescence staining, cryostat sections were incubated with anti-F4/80, anti-CD11b, anti-Gr1, anti-CD86 (Santa Cruz Biotechnology, TX, USA), anti-CD206 antibodies (BD Pharmingen, San Diego, CA, USA), anti-MyD88 (Abcam, Cambridge, Cambs, UK) followed by incubation with Alexa Fluor 488-conjugated or Alexa Fluor 594-conjugated secondary antibodies (1:500; Invitrogen, Carlsbad, CA, USA). Sections were evaluated under a micro-scope (DP71, OLYMPUS, Tokyo, Japan) for bright-field and fluorescence microscopy.

### Clinical samples

Primary human HCC and adjacent nontumor liver tissue samples were obtained from HCC patients at Xinhua Hospital Affiliated to Shanghai Jiaotong University School of Medicine, with informed consent obtained from all patients. The Ethics Committee of Beijing Jiaotong University approved the use of human specimens in accordance with the Declaration of Helsinki.

### Cell lines and treatments

The RAW264.7, Hepa1–6 and L929 cells were obtained from the American Type Culture Collection (ATCC; TIB-71, VA, USA). These cells were cultured in Dulbecco’s modified Eagle’s medium DMEM containing 10% fetal bovine serum (FBS) and 1% penicillin/streptomycin at 37 °C with 5% CO_2_. BMDMs were induced by 20 ng/mL IL-4, 20 ng/mL IL-13 recombinant protein, (200 μM, Sigma-Aldrich, St. Louis, MO, USA), and 2µM Fatostatin (inhibitor of SREBP1) for 48 h.

To obtain macrophage colony-stimulating factor (mCSF) from fibroblast conditioned medium (FCM), L929 fibroblasts were cultured in DMEM medium with 10% FBS for 3 days and the supernatant FCM was collected and centrifuged at 12,000 *g* for 10 min. The centrifuged FCM was stored at −80 °C.

To obtain tumor conditioned medium (TCM) we cultured Hepa1–6 cells for 72 h to collect the supernatant, which was filtered through a 0.45 µm filter. RAW264.7 cells were treated in accordance with TCM mixed with DMEM at a ratio of 2:3.

### Isolation of monocyte-derived macrophages

8 weeks-old mice were euthanized and their femur and tibia were crushed and flushed with PBS to collect bone marrow cells using a syringe. Bone marrow cells were cultured in a DMEM medium containing 10% FBS and 20% FCM for 1 week to generate BMDMs. By the 7th day, all adherent cells had become mature macrophages (30).

### Western blot analysis

Western blot was performed as previously described. Tissue and cell extracts (30μg protein per lane) were separated by electrophoresis on a 10% SDS-PAGE gel at 65V for 45 min and 115 V for 1 h 20 min, then transferred to a PVDF membrane at 200 mA for 1 h. Membranes were blocked with 5% milk in TBST for 2 h and incubated overnight at 4 °C with the following primary antibodies: anti-MyD88, anti-CD68 (Abcam, Cambridge, Cambs, UK), anti-FASN (Affinity Biosciences, Liyang, Jiangsu, CN), anti-SREBP1 (Affinity Biosciences, Liyang, Jiangsu, CN), anti-SCD1 (Affinity Biosciences, Liyang, Jiangsu, CN), anti-STAT6 and anti-p-STAT6 antibodies (Affinity Biosciences, Liyang, Jiangsu, CN). HRP-conjugated goat anti-mouse IgG and goat anti-rabbit IgG were used as secondary antibodies. Blots were scanned using a Clinx Science Instrument. All specific bands were quantified with the ImageJ (version 1.8 RRID: SCR_003070) automated Digitizing System.

### RNA sequencing analysis

RNA-sequencing analyses were performed in DEN/HFD-induced HCC tissues from control and Lyz^MyD88−/−^ mice. Total RNA was extracted with RNeasy Mini Kit (QIAGEN, Dusseldorf, Germany), and RNA-sequencing analyses were performed on the BGISEQ-500 sequencer platform by BGI (Shenzhen, China). Stats package and plots with ggplot2 (RRID: SCR_014601) package in R (version 3.5) were used in principle component analysis. The raw transcriptomic reads were mapped to C57BL/6 genome using HISAT40/Bowtie241 tools after removing adaptor sequences, reads containing polyN sequences, and low-quality reads. Normalization was performed and RESM software was used. Significantly differentially expressed genes (DEGs) were identified by setting padj <0.05, and the absolute value of log2 Ratio ≤ 0.5. The KEGG (Kyoto Encyclopedia of Genes and Genomes) enrichment analysis was performed by using phyper in R. All data mining, and figure presentation were conducted on the Dr Tom network platform of BGI (http://report.bgi.com).

### Single-cell RNA sequencing analysis

Samples of human HCC were analyzed by single-cell RNA sequencing (scRNA-seq), The scRNA-seq approach utilized in this study was previously described in detail (31).

### Public database analysis

Gene expression data (GSE190967 profiling data) were downloaded as raw signals from the Gene Expression Omnibus (GEO, https://www.ncbi.nlm.nih.gov/geo/), analyzed using the Geo2R function from NCBI. Pearson’s correlation analysis was performed on CD68 and MyD88 or SREBP1 and CD163. Gene expression of HCC was obtained from published gene expression profiles included in the TCGA dataset (https://www.cancer.gov/). Survival analysis was performed with MyD88, CD163 and SREBP1 gene expression bifurcated by the spline method to group into low vs high. Overall survival was calculated by the Kaplan-Meier method. Differences were analyzed by the log-rank test.

### Oxygen consumption rate

Cellular mitochondrial function was measured by using a Seahorse Bioscience XF96 Extracellular Flux Analyzer according to the manufacturer’s instructions for the Seahorse XF Cell Mito Stress Test Kit (Seahorse Bioscience, USA). Briefly, 2 × 10^4^ BMDMs were seeded into 96-well cell culture XF microplates and gave treatment for further testing. Cellular mitochondrial function was measured with separative injections of four compounds 1.5 μM Oligomycin, 1.5 μM carbonyl cyanide p-(trifluoromethoxy) phenylhydrazone (FCCP), and combination of 2.5 μM antimycin A and 2.5 μM rotenone (RAA). Finally, the results were normalized by cell numbers.

### Quantitative real-time polymerase chain reaction

Total RNA was isolated respectively from frozen liver tissues or cells by TRIzol reagent (Invitrogen, USA), and 1μg total RNA was converted to cDNA using the RT-PCR Kit (Tiangen Biotech, Beijing, CN) according to the manufacturer’s instructions qPCR was performed using the SYBR Premix ExTaqTM Kit (Tiangen Biotech, Beijing, CN). The primer sequences are listed in **Supplementary Table 1**. Data were analyzed using the 2^-ΔΔCt^ method and normalized to β-actin expression.

### Statistical analysis

All data were expressed as the mean ± SEM and analyzed using GraphPad Prism V8.0 software. Differences between the two groups were compared using two-tailed unpaired Student’s t-test analysis. Two-way ANOVA was used for multiple comparisons. *p* < 0.05 was considered statistically significant.

## Results

### MyD88 in tumor-associated macrophages was activated in HCC

**Figure 1:**
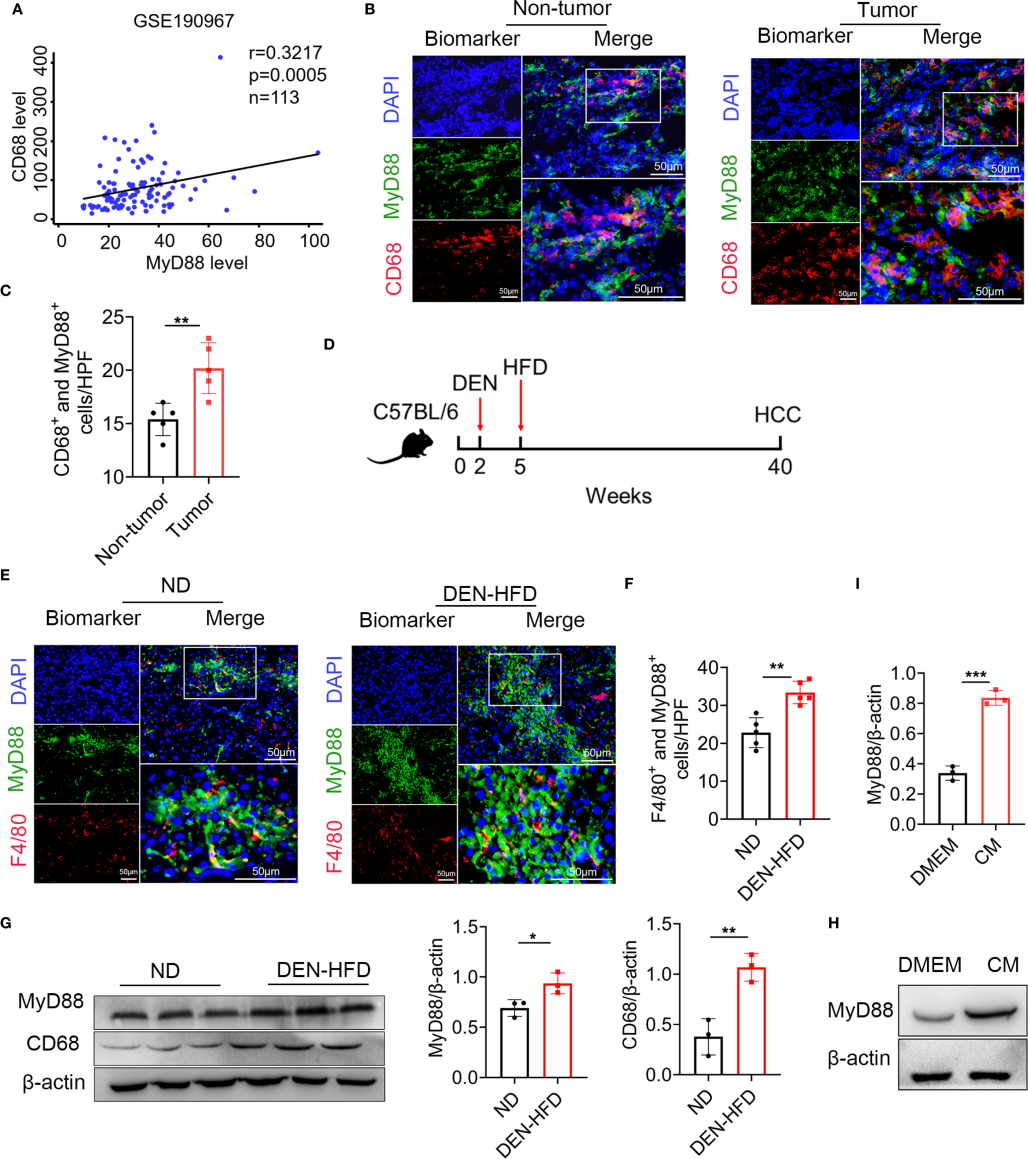
MyD88 in tumor-associated macrophages (TAMs) was upregulated in HCC. **(A)** Spearman’s correlation analysis showed correlations among CD68 and MyD88 in the liver dataset GSE190967. **(B)** Double staining of MyD88 and CD68 in tumor tissues and adjacent paracancerous tissues from HCC samples. Scale bar, 50 μm. **(C)** Statistical analysis HPF: high power field. **p<0.01. **(D)** Schematic representation of the DEN/HFD induced liver cancer model. **(E)** Double staining of F4/80 and MyD88 in normal and DEN-HFD-induced mouse HCC samples. Scale bar, 50 μm. **(F)** Statistical analysis HPF: high power field. **p<0.01. **(G)** The protein levels of MyD88 in liver tissues were determined. The densities of proteins were quantified using densitometry. Proteins were normalized to β-actin. *p<0.05, **p<0.01. **(H)** MyD88 expression levels was determined in macrophage cell line RAW264.7 after stimulation using tumor culture supernatant. **(I)** The densities of proteins were quantified using densitometry. Proteins were normalized to β-actin. *** *p* < 0.001.

We investigated the activation of MyD88 in TAMs in HCC. The GEO database analysis revealed a positive correlation between CD68 and MyD88 expression in HCC (**Figure 1A**). We also collected tissue samples from HCC patients and conducted immunofluorescence of CD68 and MyD88; MyD88 was upregulated in macrophages. Double immunofluorescence staining of CD68 and MyD88 confirmed that MyD88 expression was upregulated in macrophages in HCC tissues from patients. (**Figures 1B, C**).

We utilized the DEN-HFD-induced HCC mouse model (**Figure 1D**). We conducted immunofluorescence analysis, which revealed that the F4/80^+^ macrophages in HCC tissues expressed higher levels of MyD88 (**Figures 1E, F**). These results were consistent with the Western blot data, indicating that CD68 and MyD88 protein levels were significantly upregulated in HCC tissues (**Figure 1G**). Moreover, we treated Raw264.7 cells with a conditioned medium (CM) prepared from the Hepa1–6 liver carcinoma cell line and observed a significant upregulation of MyD88 (**Figures 1H, I**). Taken together, these results demonstrated that MyD88 activation in TAMs is associated with developing HCC.

### MyD88 deficiency in myeloid cells attenuated DEN/HFD-induced NAFLD-related hepatocarcinogenesis

**Figure 2:**
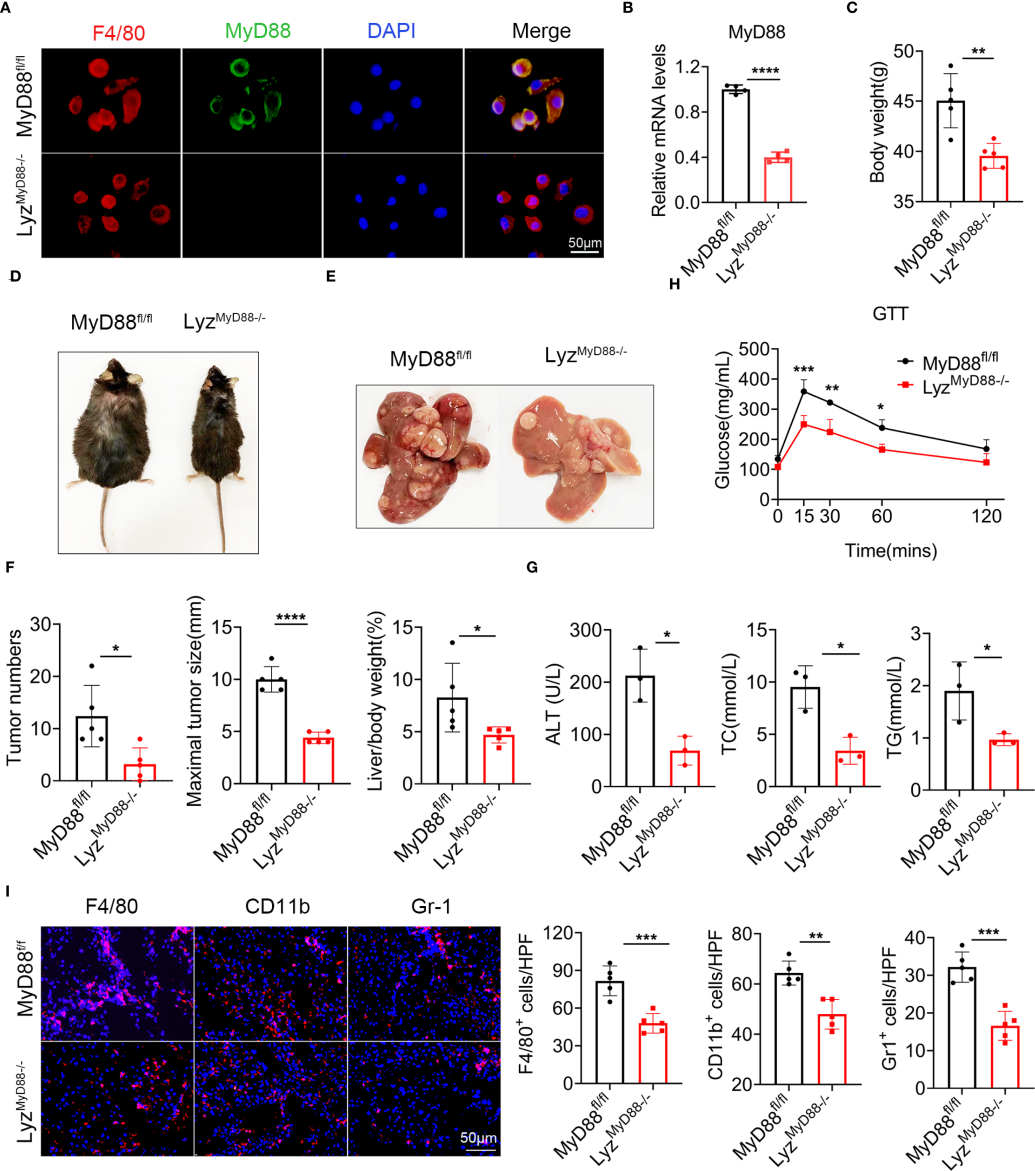
MyD88 deficiency in myeloid cells attenuated DEN/HFD-induced NAFLD-related hepatocarcinogenesis. **(A)** Double staining of F4/80 and MyD88 in primary macrophages from MyD88^fl/fl^ and Lyz^MyD88−/−^ mice, ****p<0.0001. Scale bar, 50 μm. **(B)** The mRNA expression levels of MyD88 in myeloid cells from MyD88^fl/fl^ and Lyz^MyD88−/−^ mice. **(C)** Body weight at the end of diet. **(D)** Representative images of MyD88^fl/fl^ and Lyz^MyD88−/−^ mice at the end of a high-fat diet. **(E)** Representative images of the livers from Lyz^MyD88−/−^ mice and control mice. **(F)** Tumors number, maximal tumor size, liver-to-body ratio statistics in MyD88^fl/fl^ and Lyz^MyD88−/−^ mice. *p<0.05, **** *p* < 0.0001. **(G)** ALT, TC, TG in MyD88^fl/fl^ and Lyz^MyD88−/−^ mice serum. *p<0.05. **(H)** Glucose tolerance of MyD88^fl/fl^ and Lyz^MyD88−/−^ mice *p<0.05, **p<0.01, *** *p* < 0.001. **(I)** Representative staining of F4/80, Gr1 and CD11b in liver tissues (Scale bar, 50μm) and statistical analysis. **p<0.01, *** *p* < 0.001.

To explore the role of MyD88 in macrophages, we conditionally deleted MyD88 in murine myeloid cells as previously reported (32). We crossed mice carrying the loxP-flanked MyD88 allele with Lysm-cre mice to achieve MyD88 ablation specifically in myeloid cells (Lyz^MyD88−/−^) mice. The Lysm-cre-MyD88^fl/fl^ (Lyz^MyD88−/−^) mice were identified by PCR. We confirmed the absence of MyD88 in myeloid cells from the Lyz^MyD88−/−^ mice by double immunofluorescence staining of CD68 and MyD88 (**Figure 2A**). The mRNA level of MyD88 in Lyz^MyD88−/−^ mice livers decreased significantly compared with MyD88^fl/fl^ mice (**Figure 2B**).

To investigate the role of MyD88 in myeloid cells in NAFLD-related HCC, we administered an intraperitoneal injection of DEN to Lyz^MyD88−/−^ and MyD88^fl/fl^ mice, then fed them an HFD beginning at 6 weeks of age. We monitored liver tumorigenesis for 10 months and found that Lyz^MyD88−/−^ mice had significantly reduced body weight gain (**Figures 2C, D**). All control mice developed liver tumors within 40 weeks. However, Lyz^MyD88−/−^ mice showed obvious resistance to liver cancer development. Myeloid cell-specific MyD88 deletion significantly decreased the number and size of HCC tumors at 40 weeks after DEN administration. Lyz^MyD88−/−^ mice also had lower liver/body weight. (**Figures 2E, F**). Serum alanine aminotransferase (ALT), triglyceride (TG), and total cholesterol (TC) were significantly decreased in DEN/HFD-treated Lyz^MyD88−/−^ mice compared to MyD88^fl/fl^ mice (**Figure 2G**). Lyz^MyD88−/−^ mice exhibited an improved glucose tolerance compared with MyD88^fl/fl^ mice (**Figure 2H**). Furthermore, MyD88 deletion in macrophage significantly decreased the infiltration of F4/80^+^ macrophages, Gr-1^+^ neutrophils, and CD11b^+^ monocytes in NAFLD-related HCC (**Figure 2I**). In addition, the infiltration of CD4^+^ and CD8^+^ T cells was slightly decreased in Lyz^MyD88−/−^ mice compared to MyD88^fl/fl^ mice (**Supplementary Figure S1A**). Collectively, these data suggest that myeloid cell-specific MyD88 deficiency attenuates the development of NAFLD-related HCC and macrophages may play a more essential role in this process.

### MyD88 deficiency in myeloid cells attenuated liver fat accumulation

**Figure 3:**
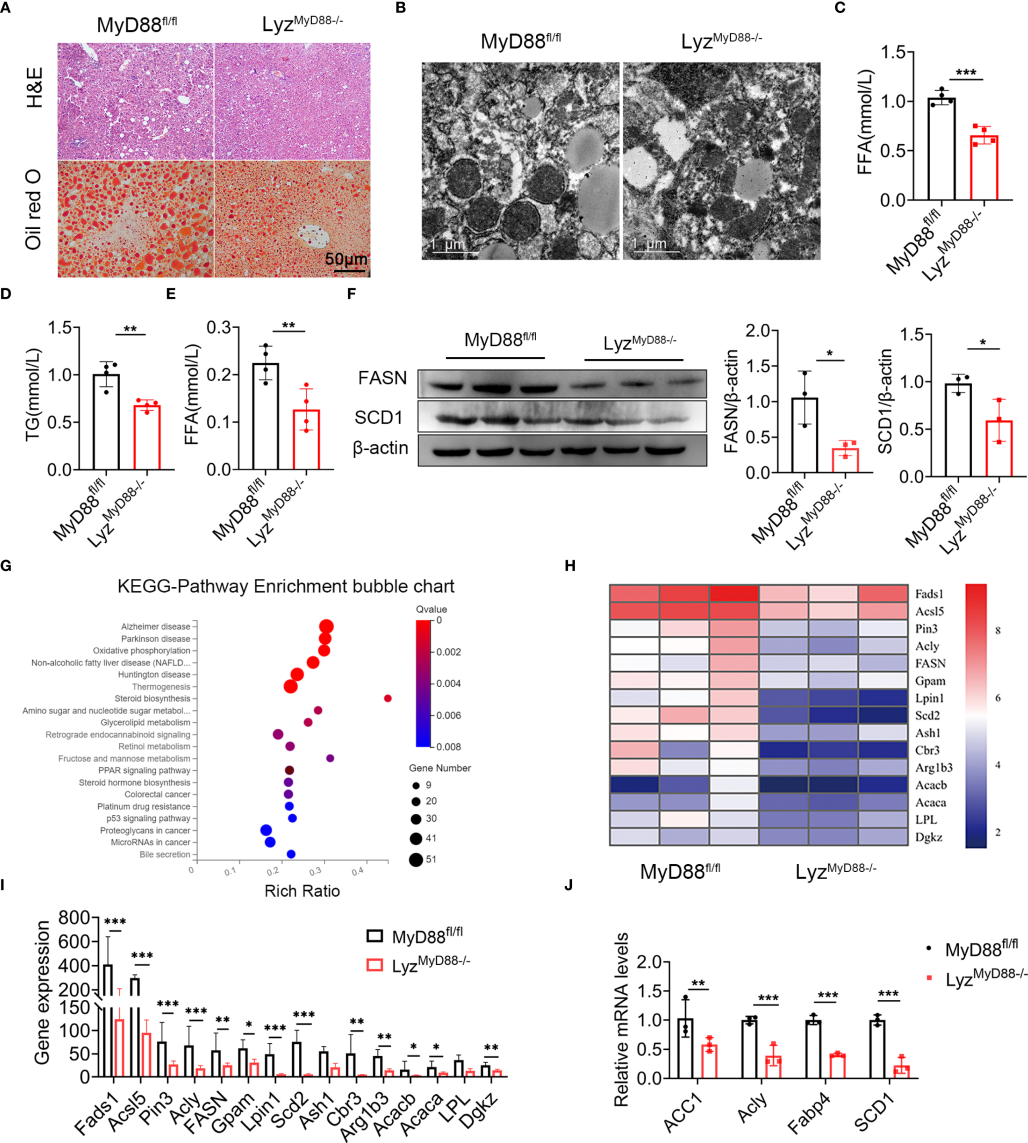
MyD88 deficiency in myeloid cells attenuated fat accumulation. **(A)** H&E and Oil Red O staining of liver tissue from DEN-HFD-induced MyD88^fl/fl^ and Lyz^MyD88−/−^ mice. **(B)** Transmission electron micrographs of liver tissue from DEN-HFD-induced MyD88^fl/fl^ and Lyz^MyD88−/−^ mice. **(C)** The level of FFA in liver tissue from DEN-HFD-induced MyD88^fl/fl^ and Lyz^MyD88−/−^ mice. *** *p* < 0.001. **(D)** The level of TG in liver tissue from DEN-HFD-induced MyD88^fl/fl^ and Lyz^MyD88−/−^ mice. **p<0.01. **(E)** FFA levels in the blood of DEN-HFD-induced MyD88^fl/fl^ and Lyz^MyD88−/−^ mice. **p<0.01. **(F)** The protein levels of SCD1 in liver tissue of DEN-HFD MyD88^fl/fl^ and Lyz^MyD88−/−^ mice. The densities of proteins were quantified using densitometry. Proteins were normalized to β-actin. *p < 0.05. **(G)** Enrichment analysis of liver tissue RNA sequencing KEGG signaling pathway in DEN-HFD MyD88^fl/fl^ and Lyz^MyD88−/−^ mice. **(H)** Heat map of metabolism-related genes by RNA sequencing of liver tissue from DEN-HFD MyD88^fl/fl^ and Lyz^MyD88−/−^ mice. **(I)** Metabolism-related gene expression in liver tissues. *p<0.05, **p<0.01, *** *p* < 0.001. **(J)** The mRNA levels of ACC1, Acly, Fabp4 and SCD1 in the liver tissues from MyD88^fl/fl^ and Lyz^MyD88−/−^ mice. **p<0.01, *** *p* < 0.001.

To investigate the effect of MyD88 in myeloid cells on fat accumulation in mice, H&E and Oil red O staining revealed that DEN/HFD-treated Lyz^MyD88−/−^ mice had less fat accumulation compared with MyD88^fl/fl^ mice (**Figure 3A**). Moreover, we observed more mitochondria and fat vacuoles in MyD88^fl/fl^ mice with electron microscope (**Figure 3B**). We also found Lyz^MyD88−/−^ mice had lower serum levels of free fatty acids (FFA) and TG compared with MyD88^fl/fl^ mice (**Figures 3C–E**). We further examined lipogenesis gene expression in HCC tissues. As shown in **Figure 3F**, FASN and SCD1 protein levels were significantly downregulated in HCC tissues from Lyz^MyD88−/−^ mice. Furthermore, a protein-coding mRNA-seq analysis of liver tissues from DEN/HFD-treated Lyz^MyD88−/−^ mice and MyD88^fl/fl^ was conducted, and 344 differentially expressed genes were identified, including 58 upregulated and 286 downregulated genes. The subsequent analysis revealed that MyD88 deficiency in macrophages changed various metabolic-related signal pathways and various metabolic- related enzymes (**Figures 3G–I**). Therefore, qPCR analysis was conducted to detect the lipid metabolic-related gene expression, which decreased in Lyz^MyD88−/−^ mice (**Figure 3J**). Collectively, these findings suggest that macrophage-specific MyD88 deficiency attenuates the development of HFD-induced NAFLD.

### Macrophage-specific deletion of MyD88 inhibits macrophage M2 polarization

**Figure 4:**
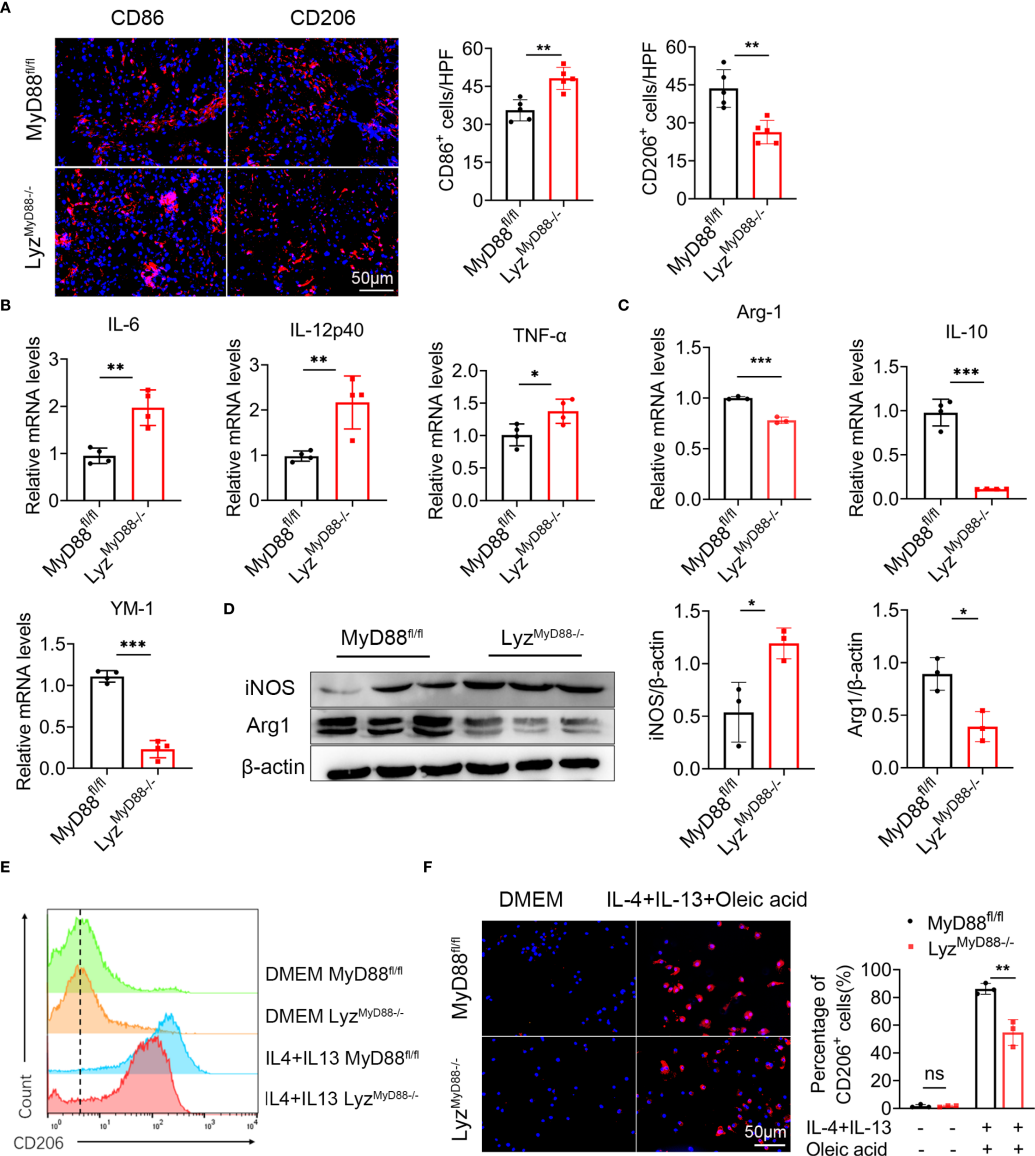
Macrophage-specific deletion of MyD88 inhibits macrophage M2 polarization. **(A)** Liver tissue of DEN-HFD MyD88^fl/fl^ and Lyz^MyD88−/−^ mice were stained for M1 macrophage polarization marker CD86 and M2 macrophage polarization marker CD206 (Scale bar, 50μm) and statistical analysis. **p<0.01. **(B)** The mRNA levels of IL-6, IL-12p40, TNF-α in the liver tissues of MyD88^fl/fl^ and Lyz^MyD88−/−^ mice were measured. *p<0.05, **p<0.01. **(C)** The mRNA levels of IL-10, Arg1, YM1 in the liver tissues from MyD88^fl/fl^ and Lyz^MyD88−/−^ mice. **p<0.01, *** *p* < 0.001. **(D)** The protein levels of iNOS and Arg1 in liver tissues of MyD88^fl/fl^ and Lyz^MyD88−/−^ mice. The densities of proteins were quantified using densitometry. Proteins were normalized to β-actin. *p < 0.05. **(E)** Induction of M2 macrophages using oleic acid, IL-4, and IL-13. Detection of M2 macrophage marker CD206 by flow cytometry. **(F)** Induction of M2 macrophages using oleic acid, IL-4, IL-13, and immunofluorescence double staining (Scale bar, 50μm) and statistical analysis. **p<0.01.

To further explore how MyD88 in macrophages affects nonalcoholic fatty liver related liver cancer, we analyzed the number of CD206^+^ (an M2 marker) and CD86^+^ (an M1 marker) macrophages in liver tumor tissues and found that Lyz^MyD88−/−^ mice exhibited an increase in the M1-like phenotype and a reduction in the M2-like phenotype compared to those in control mice (**Figure 4A**). Furthermore, MyD88 deletion in macrophages also resulted in an upregulation of M1-related genes (IL-6, IL-12p40 and TNF-α) and a downregulation of M2-related genes (Arg-1, IL-10 and YM-1) (**Figures 4B, C**). Higher expression of iNOS (an M1 marker) and the lower expression of Arg1 (an M2 marker) in HCC tissues where MyD88 was ablated in macrophages was also observed (**Figure 4D**). To further confirm these observations, we carried out an *in vitro* analysis. Bone marrow-derived macrophages (BMDMs) isolated from Lyz^MyD88−/−^ mice and control mice were stimulated with an exogenous fatty acid, IL-4 and IL-13 and were induced to M2 phenotype. A consistent conclusion was also achieved in FACS analysis (**Figure 4E**). Moreover, CD206 expression in macrophages was analyzed by immunofluorescence staining and results showed that MyD88 deficiency in macrophages attenuated macrophage M2 polarization compared with control (**Figure 4F**). Therefore, these results indicated that MyD88 deletion in macrophages reprogrammed the macrophages to inhibit M2 polarization.

### Macrophage-specific deletion of MyD88 inhibits macrophage metabolism

**Figure 5:**
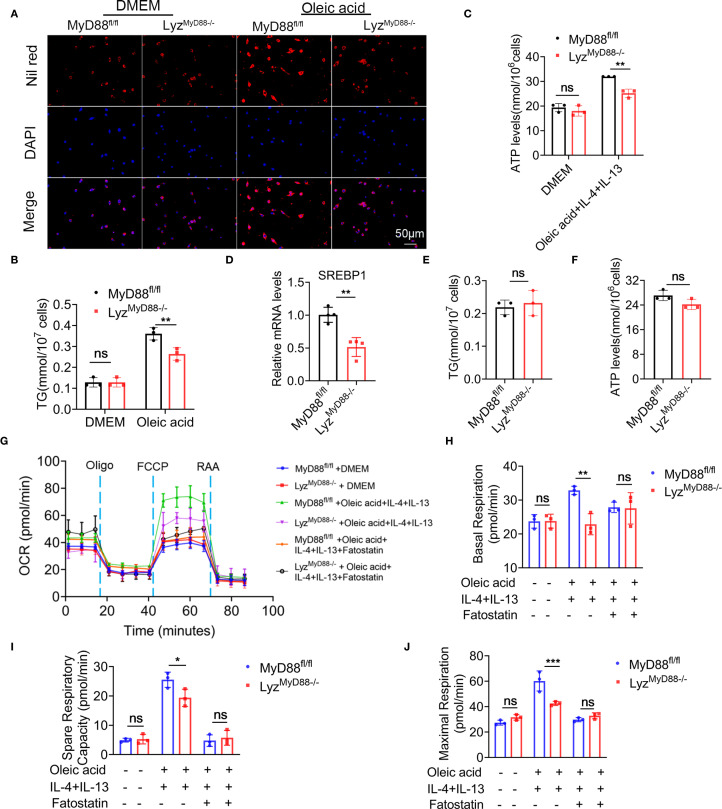
Macrophage-specific deletion of MyD88 inhibits macrophage metabolism. **(A)** Nil red staining of oleic acid-stimulated primary macrophages from MyD88^fl/fl^ and Lyz^MyD88−/−^ mice. **(B)** TG levels in Oleic acid stimulated primary macrophages. **p<0.01. **(C)** ATP levels in Oleic acid, IL-4 and IL-13 induced M2 macrophages. **p<0.01. **(D)** The RNA levels of SREBP1 in mice liver tissue. ** *p* < 0.01. **(E, F)** TG and ATP levels in SREBP1 inhibitor Fatostatin and oleic acid treated macrophages. **(G–J)** BMDMs were treated with Oleic acid, IL-4, IL-13 and SREBP1 inhibitor Fatostatin, and its cellular mitochondrial function was measured with Cell Mito stress test kit. The Oxygen Consumption Rate (OCR) profile, basal respiration, spare respiratory capacity and maximal respiration were quantified. *p<0.05, **p<0.01, *** *p* < 0.001.

One of the essential features of macrophage polarization is metabolic reprogramming. We were curious to know if macrophage metabolism is affected by MyD88. Primary macrophages were stimulated with oleic acid and stained with Nile red to investigate this. Macrophages from Lyz^MyD88−/−^ mice showed a significant decrease in the accumulation of lipid droplets. Moreover, TG level was also notably reduced in macrophages from Lyz^MyD88−/−^ mice (**Figures 5A, B**).

To detect ATP levels, M2 macrophages were induced with oleic acid, IL-4 and IL-13. It was found that macrophages lacking MyD88 had a significantly lower ATP level (**Figure 5C**). SREBP1 is a key enzyme in Toll-like receptor metabolism. The mRNA level of SREBP1 was significantly decreased in liver tissues from Lyz^MyD88−/−^ mice compared with control mice (**Figure 5D**). indicating that MyD88 promotes macrophage metabolism via SREBP1. To test this hypothesis, macrophages were treated with the SREBP1 inhibitor Fatostatin. As shown in **Figures 5E, F**, adding Fatostatin to oleic acid, IL-4, and IL-13-induced macrophage abolished TG and ATP levels in MyD88^fl/fl^ mice (**Figures 5E, F**).

To further explore the role of MyD88 in regulating macrophage metabolism, we measured the oxygen consumption rate (OCR) in macrophages using Seahorse X96. MyD88-deficient macrophages exhibited decreased basal respiration, maximal respiration, and respiratory capacity after induction of M2 macrophage polarization using oleic acid, IL-4, and IL-13 compared to control (**Figures 5G–J**). However, there was no obvious difference before M2 induction. Furthermore, adding Fatostatin to oleic acid, IL-4, and IL-13-induced macrophage abolished basal respiration, maximal respiration, and spare respiratory capacity in macrophages from MyD88^fl/fl^ mice. Therefore, MyD88 in macrophages regulates metabolism partly through SREBP1. The deletion of MyD88 in macrophages inhibits macrophage M2 polarization possibly through attenuating macrophage metabolism.

### Macrophage-specific deletion of MyD88 inhibits macrophage M2 polarization by inhibiting SREBP1/STAT6 pathway

**Figure 6:**
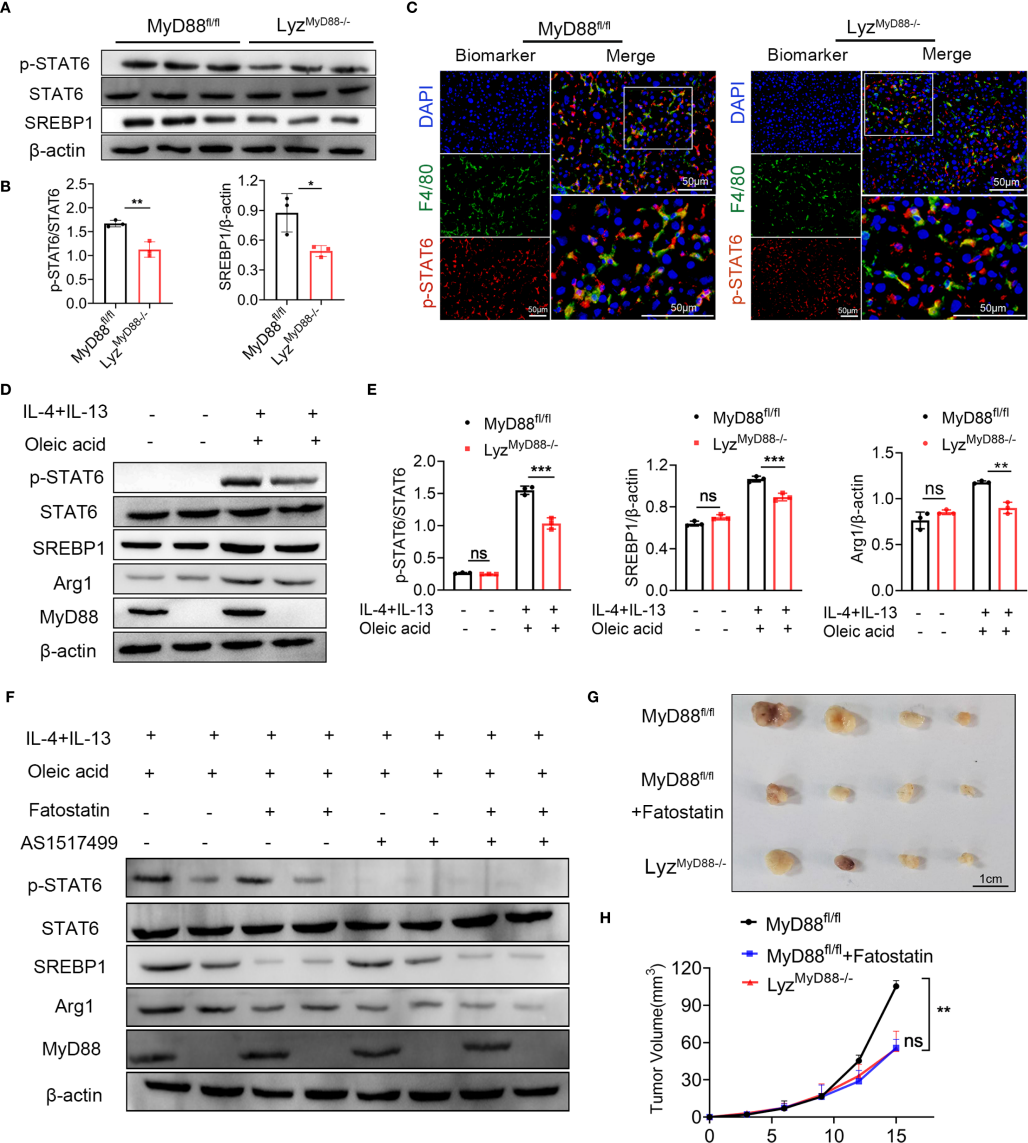
MyD88 promotes macrophage M2 polarization through SREBP1/STAT6 pathway. **(A, B)** The protein levels of SREBP1, STAT6, p-STAT6 in liver tissues of MyD88^fl/fl^ and Lyz^MyD88−/−^ mice. The densities of proteins were quantified using densitometry. p-STAT6 was normalized to STAT6, SREBP1 was normalized to β-actin. *p < 0.05, **p < 0.01. **(C)** Representative double-staining of p-STAT6 and F4/80 in colon tumors (Scale bars: 50μm). **(D, E)** The protein levels of SREBP1, STAT6, p-STAT6 and Arg1 in M2 macrophages. The densities of proteins were quantified using densitometry. p-STAT6 was normalized to STAT6, SREBP1 and Arg1were normalized to β-actin. **p<0.01, *** *p* < 0.001. **(F)** The protein levels of SREBP1, STAT6, p-STAT6 and Arg1 in M2 macrophages. **(G)** Representative images of Hepa1–6 transplanted tumors in MyD88^fl/fl^ and Lyz^MyD88−/−^ mice. **(H)** Growth curves of Hepa1–6 transplanted tumors in MyD88^fl/fl^ and Lyz^MyD88−/−^ mice. **p<0.01.

The expression of the STAT family members has been associated with macrophage polarization, with higher levels of STAT6 in M2 macrophages. To elucidate the mechanism by which MyD88 affects macrophage polarization, we analyzed HCC tissues and macrophages using Western blot. Our findings showed that Lyz^MyD88−/−^ mice had a lower phosphorylated STAT6 (p-STAT6) level compared with MyD88^fl/fl^ mice. Lyz^MyD88−/−^ mice also had a lower expression of SREBP1 compared with control mice (**Figures 6A-B**). Further immunofluorescence analysis showed that the expression of p-STAT6 in macrophages was reduced in Lyz^MyD88−/−^ mice with HCC (**Figure 6C**).

In line with the animal experiment results, oleic acid, IL-4 and IL-13-induced M2 macrophages significantly upregulated the expression of p-STAT6, SREBP1 and Arg1. However, MyD88 deletion in macrophages showed lower levels of p-STAT6, SREBP1 and Arg1 (**Figures 6D, E**). To further verify whether MyD88 affects macrophage polarization through SREBP1/STAT6 signaling, macrophages were treated with the STAT6 inhibitor AS1517499 and SREBP1 inhibitor Fatostatin. As shown in **Figures 6F**, the addition of AS1517499 and Fatostatin to oleic acid, IL-4 and IL-13-induced macrophages, abolished p-STAT6 and SREBP1 levels. MyD88 deficiency in macrophages had a lower Arg1 level, confirming that MyD88 promotes macrophage M2 polarization mainly by activating SREBP1/STAT6 signaling.

To further investigate the role of SREBP1 in liver tumor development, Lyz^MyD88−/−^ mice and MyD88^fl/fl^ mice with established Hepa1–6 tumors. MyD88^fl/fl^ mice were treated with an SREBP1 inhibitor, demonstrating a significant anti-tumor effect (**Figures 6G, H**). Together, these results indicated that deleting MyD88 inhibits macrophage M2 polarization, possibly due to the deactivation of STAT6 and SREBP1 signals in macrophages.

### MyD88 and SREBP1 expression was upregulated in macrophages and was associated with shorter survival in patients with HCC

**Figure 7:**
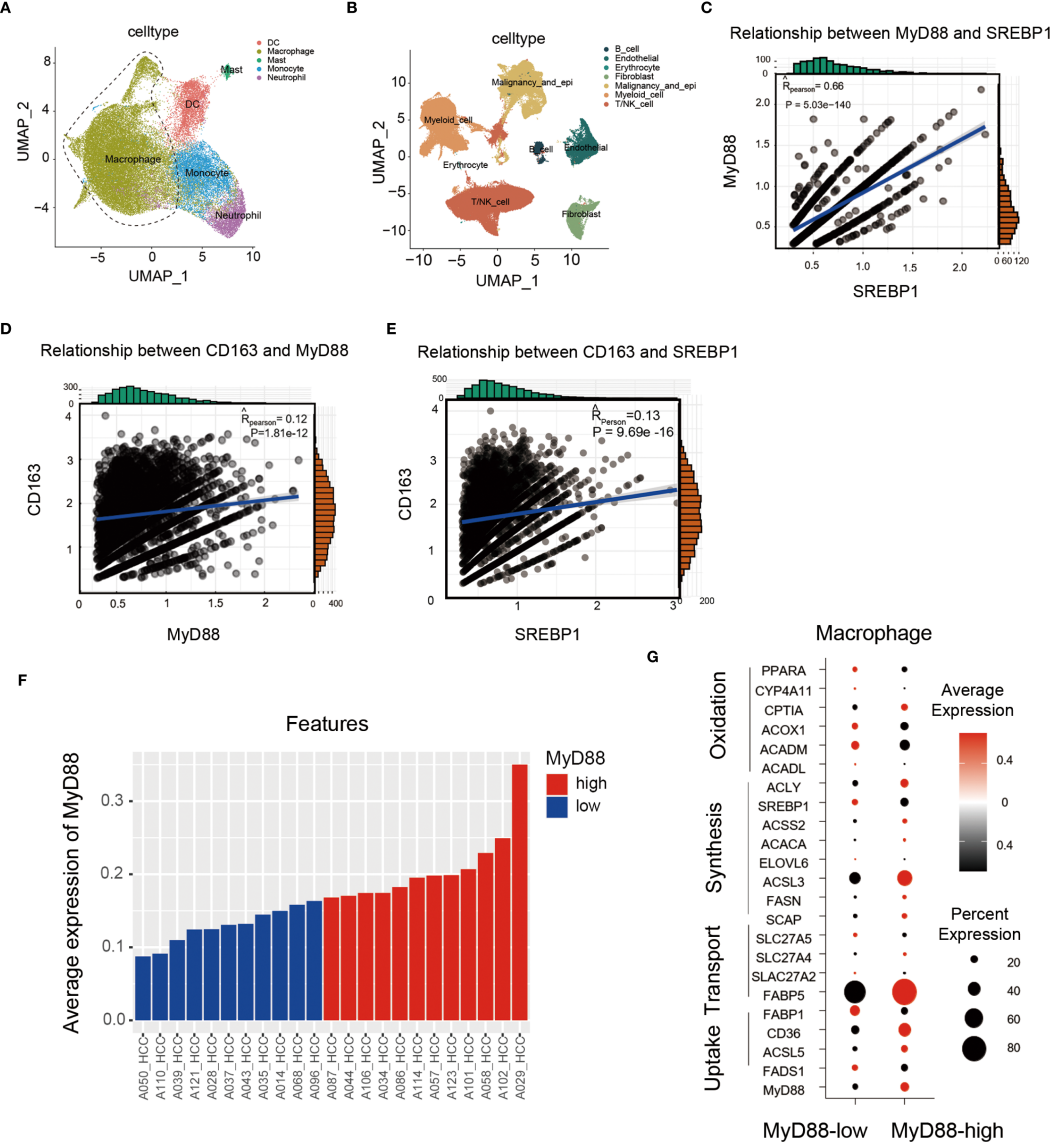
In HCC patients, MyD88 and SREBP1 expression was elevated in M2 macrophages, and MyD88 was associated with macrophage metabolism. **(A, B)** Scatterplots of immune cell types in HCC patients. **(C)** Correlation analysis of MyD88 with SREBP1 in CD163^+^ cells. **(D)** Correlation analysis of MyD88 with CD163 in macrophages. **(E)** Correlation analysis of SREBP1 with CD163 in macrophages. **(F, G)** Expression analysis of MyD88 and metabolism-related genes in macrophages.

We collected liver tissues from patients with HCC for single-cell sequencing (31). Our analysis of macrophage subpopulations showed a positive correlation between SREBP1 and MyD88 expression in macrophages, and CD163 was also positively related to the expression of MyD88 and SREBP1 (**Figures 7A–E**). We divided the patients into two groups based on their MyD88 expression levels and discovered that the high MyD88 expression group had more upregulated genes related to fatty acid metabolism (**Figures 7F, G**). Overall, our single-cell sequencing results suggest that MyD88 and SREBP1 may positively regulate M2 macrophage polarization, and the macrophage metabolism was more dynamic in patients in the MyD88 high-expression group.

**Figure 8:**
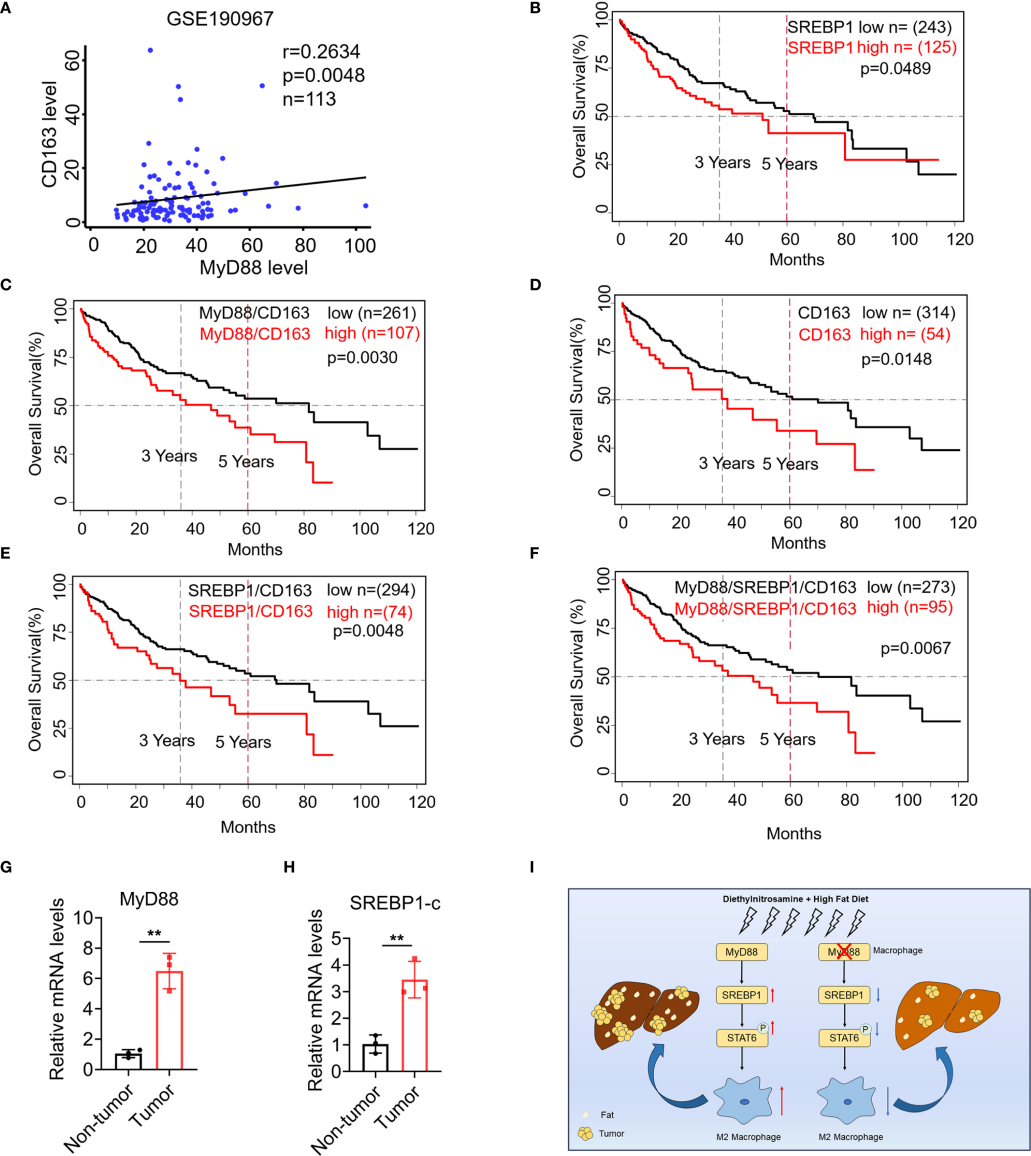
SREBP1 expression was increased in macrophage of HCC and was associated with shorter survival of patients with HCC. **(A)** Spearman’s correlation analysis showed correlations among CD163 and MyD88 in the liver dataset GSE190967. **(B–F)** Kaplan-Meier curve for survival in the HCC TCGA dataset with MyD88, CD163 and SREBP1, combined MyD88/CD163/SREBP1 gene expression bifurcated by the spline method to group into low vs high. Three years and five years are indicated as dotted lines. **(G, H)** The mRNA levels of MyD88 and SREBP1 in the liver tissues of HCC patients. **p<0.01. **(I)** Schematic of MyD88 signaling in macrophages in the model of HFD-related HCC.

Furthermore, we analyzed the publicly available GEO dataset GSE190967, and found that CD163 (a macrophage marker) and MyD88 were positively correlated through correlation analysis. (**Figure 8A**). Additionally, by using RNA-seq data gathered from the TCGA database (http://cancergenome.nih.gov/), we found that patients with high levels of CD68, MyD88 or SREBP1 survived significantly worse than those with low levels (**Figures 8B–E**). A combined high expression of CD68/MyD88/SREBP1 also led to significantly impaired overall survival compared to that of patients with low CD68/MyD88/SREBP1 expression (**Figure 8F**). Furthermore, we examined the expression of MyD88 and SREBP1-c in liver tissue from HCC patient. Consistent with before results, there was a higher expression of MyD88 and SREBP1 in tumor compared with non-tumor tissue (**Figures 8G, H**). Together, these data suggest that upregulation of MyD88 and SREBP1 in macrophages is associated with a poor prognosis in HCC.

## Discussion

This study investigated the role of MyD88 in macrophages during the progression from NAFLD to HCC. Our findings suggest that MyD88 deficiency in macrophages can reduce lipid accumulation in NAFLD-associated HCC. Moreover, MyD88 in macrophages promotes the expression of SREBP1 and enhances macrophage M2 polarization. This is achieved by activating the SREBP1/STAT6 pathway, which supports hepatocarcinogenesis (**Figure 8I**).

Previous studies have highlighted the critical role of MyD88 in obesity-related NAFLD. Complete deletion of MyD88 in HFD-fed mice produced obesity, insulin resistance, and hepatic steatosis (33, 34). The MyD88 protein is present in various immune and non-immune cells. Recent studies suggest that non-immune MyD88 proteins perform different functions depending on the cell type and function. If MyD88 is deleted in hepatocytes, it can lead to glucose intolerance, inflammation and insulin resistance in the liver, regardless of the person’s weight or adiposity (35). In contrast, MyD88 depletion in myeloid cells and intestinal epithelial cells reduced diet-induced obesity, systemic inflammation, and insulin resistance (21, 27). In another study, myeloid-derived MyD88 is required for HFD-induced systemic inflammation, and MyD88 deficiency in myeloid and endothelial cells improves diet-induced hyperglycemia and hyperinsulinemia (27). In this study, we found that specific deletion of MyD88 in macrophages attenuated hepatic steatosis, inflammatory cell infiltration, and adipogenesis in an HFD-induced NAFLD mouse model (**Figure 2**), suggesting a crucial role of MyD88 in myeloid cells in NAFLD.

MyD88 plays important roles in a variety of cancers. It is widely expressed in all liver cell types, including immune cells (Kupffer cells and liver dendritic cells) and non-immune cells, such as hepatocytes and HSCs (36). MyD88-deficient mice show a significant reduction in tumor incidence in DEN-induced liver cancer (22). MyD88 signaling also contributes to HBV-mediated liver carcinogenesis (37), and its elevated expression may serve as a prognostic biomarker in HCC patients (38).

Although MyD88 is widely expressed in different cells, cell type-specific differences in MyD88 function are still largely unknown. Mohs et al. found that TLRs signal through MyD88 in non-parenchymal liver cells is required for carcinogenesis during chronic liver injury. However, hepatocyte-specific MyD88 deletion did not affect HCC progression (39). Recently, our study has shown that MyD88 in myofibroblasts promotes HCC by enhancing the secretion of CCL20 and aerobic glycolysis (25). Consistent with the above research, we found MyD88 in macrophages also promotes HCC development.

Tumor-associated macrophages are an important component of the tumor microenvironment. TAMs are present in most tumors, and high infiltration of TAMs is associated with poor prognosis of tumor patients (40). In patients with HCC, the aggregation of liver macrophages is significantly associated with a low survival rate (41). TAMs can promote tumor growth and invasion, support neoangiogenesis, suppress adaptive immunity against cancer and inhibit T cell recruitment, thereby promoting tumor progression (42). In addition, TAMs also play a pivotal role in HCC therapy and prognosis (43). In our study, we found that MyD88 in macrophages promotes macrophage M2 polarization via SREBP1/STAT6 and enhances the progression of NAFLD to HCC. However, it is yet to be determined if MyD88 has any role in NAFLD-related HCC via other mechanisms. Given that glucose tolerance and hepatic lipid accumulation may arise from changes in food intake in mice, this issue warrants further investigation in future studies.

SREBP is a regulatory element binding protein situated in the endoplasmic reticulum membrane, which functions as a transcription factor in the biosynthesis of sterol and regulates lipid metabolism (44). SREBP1 is highly expressed in various malignancies, such as colorectal cancer, liver and bladder cancer. It involves multiple pathogenic processes including endoplasmic reticulum stress, inflammation, obesity and non-alcoholic fatty liver disease et al (45–47). Our research has shown that SREBP1 expression was upregulated during the process of macrophage M2 polarization in **Figure 6D**. Using an SREBP1 inhibitor has significantly inhibited the polarization of M2 macrophages. This confirms the crucial role of SREBP1 in macrophage M2 polarization, which is consistent with recent investigations that have demonstrated that SREBP1 regulates the metabolism of tumor-promoting macrophages (48). Furthermore, our research reveals that MyD88 deletion in macrophages has lower levels of p-STAT6, SREBP1 and Arg1 (**Figure 6B**). And the addition of inhibitors to macrophages, abolished p-STAT6 and SREBP1 levels, significantly decreased the Arg1 level, indicated that MyD88 promotes macrophage M2 polarization mainly by activating SREBP1/STAT6 signaling (**Figure 6C**). Nevertheless, whether MyD88 affects macrophage M2 polarization through other pathways is yet to be determined. What’s more, SERBP1 inhibitor significantly suppressed the growth of mouse xenografts (**Figures 6G, H**). However, this inhibitor also could inhibit metabolism of other cells such as hepatocytes or tumor cells, which can affect the tumor growth. SREBP1c modulates insulin sensitivity in hepatocytes and adipocytes; its inhibition might exacerbate insulin resistance in certain contexts (49). The potential mechanisms of SREBP1 inhibitors in tumor treatment need to be further clarified.

In conclusion, MyD88 in macrophages has a promotional role in NAFLD-associated HCC. Specifically, MyD88 promotes macrophage M2 polarization, which enhances the progression of NAFLD to HCC by activating the SREBP1/STAT6 pathway. MyD88 in macrophages may be a potential therapeutic and/or preventive target for NAFLD-associated HCC.

## Data Availability

The datasets presented in this study can be found in online repositories. The names of the repository/repositories and accession number(s) can be found in the article/**Supplementary Material**.
