# Supplementary material for: MyD88 orchestrates fatty acid metabolism in tumor-associated macrophages and non-alcoholic fatty liver disease-related hepatocarcinogenesis
Source: Front Immunol. 2025 Sep 23;16:1589255. doi: 10.3389/fimmu.2025.1589255 (PMC12500666; doi:10.3389/fimmu.2025.1589255)
Supplement: Supplementary file 1 [file DataSheet1.pdf]

Supplementary materials

Table S1. Real-time PCR primer sequences.

Gene (mouse)	primer sequence
Cre F	GATCTCCGGTATTGAAACTCCAGC
Cre R	GCTAAACATGCTTCATCGTCGG
MyD88 F	GCTCCTCTTAGGGGCCACT
MyD88 R	CCACGTCTCACCATTGGGG
SCD1 F	TTCTTGCGATACACTCTGGTGC
SCD1 R	CGGGATTGAATGTTCTTGTCGT
Acly F	GCCAGCGGGAGCACATC
Acly R	GTTTGCAGGTGCCACTTCATC
Fabp1 F	AGGGGGTGTCAGAAATCGTG
Fabp1 R	GTCATGGTCTCCATGAGTGA
SREBP1 F	GGAGTGGGTAAACTGAGGCT
SREBP1 R	TTTGATCCCGGAAGCTCTGT
iNOS F	CGGAGATCAATGTGGCTGTG
iNOS R	GAAGGACTCTGAGGCTGTGT
IL-6 F	TTCTTGGGACTGATGCTGGT
IL-6 R	CTGTGAAGTCTCCTCTCCGG
MCP-1 F	GCTACAAGAGGATCACCAGCAG
MCP-1 R	GTCTGGACCCATTCCTTCTTGG

TNF-α F	TGAGGTCAATCTGCCCAAGT
TNF-α R	GGGGTCAGAGTAAAGGGGTC
IL-12p40 F	GACATGTGGAATGGCGTCTC
IL-12p40 R	TTATTCTGCCGTGCTTC
Arg1 F	CTCCAAGCCAAAGTCCTTAGAG
Arg1 R	AGGAGCTGTCATTAGGGACATC
IL-10 F	GCTCTTACTGACTGGCATGAG
IL-10 R	CGCAGCTCTAGGAGCATGTG
YM1 F	CTCAACCTGGACTGGCAGTA
YM1 R	CTGCTCCTGTGGAAGTGAGT
β-actin F	ACCAGTTCGCCATGGATGAC
β-actin R	TGCCGGAGCCGTTGTC

F: forward primer; R: reverse primer

Supplementary figure S1

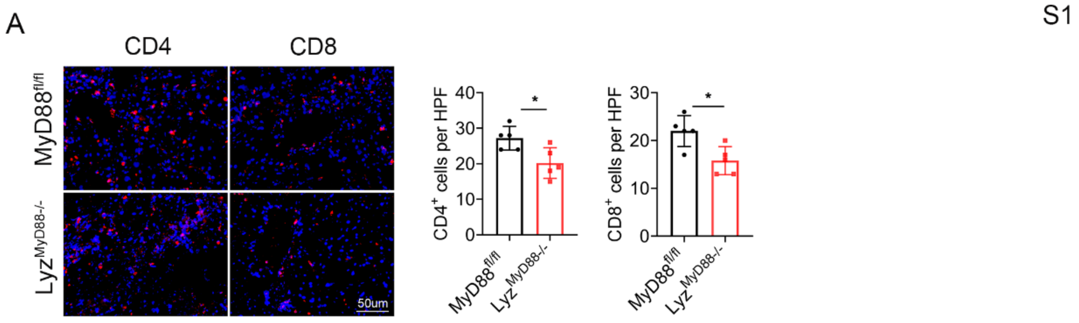

Figure S1: MyD88 deletion in macrophages decreases the infiltration of CD4$^+$ and CD8$^+$ T cells in liver from DEN/HFD-treated MyD88$^{fl/fl}$ and Lyz$^{MyD88-/-}$ mice.

A. Representative staining of CD4 and CD8 in liver tissues (Scale bar, 50μm) and statistical analysis. *$p < 0.05$.
